# Levels of S100B are raised in female patients with schizophrenia

**DOI:** 10.1186/1471-244X-13-146

**Published:** 2013-05-24

**Authors:** Kara O’Connell, Jogin Thakore, Kumlesh K Dev

**Affiliations:** 1 Molecular Neuropharmacology, Department of Physiology, School of Medicine, Trinity College Institute of Neuroscience, Trinity College Dublin, Dublin, Ireland; 2 Department of Psychiatry, School of Medicine, Trinity College Institute of Neuroscience, Trinity College Dublin, Dublin, IRELAND; 3 Neuroscience Centre, St. Vincent’s Hospital Fairview, Fairview, Dublin, Ireland

**Keywords:** S100B, Clozapine, Antipsychotics, Metabolic syndrome, Schizophrenia

## Abstract

**Background:**

The neurotrophic factor, S100B, is released primarily from astrocytes, with serum and CSF levels of S100B reported as altered in schizophrenia. However, many of these reports are contradictory. Here, serum levels of S100B in schizophrenia and influence of age, gender, medication and illness severity were examined.

**Methods:**

Serum S100B levels were measured in patients with schizophrenia treated with clozapine. Lifestyle, metabolic and illness severity parameters were correlated with S100B concentrations.

**Results:**

Data showed raised serum levels of S100B in schizophrenia female patients, but not male patients, compared to controls. Correlation analysis demonstrated a positive association between S100B serum concentrations and BMI.

**Conclusions:**

This study supports previous findings that adipocytes may contribute to S100B serum concentrations in females, in addition to astrocytes. This study also supports the hypothesis that metabolic effects of medication, lifestyle choices and the illness itself, may be contributing factors to altered levels of S100B.

## Background

S100B is a calcium-binding protein of 92 residues, with its gene located on 21q22.3 [1-3]. Within the central nervous system (CNS), S100B is thought to be a marker for astroglial activation, linking astrocyte dysfunction to schizophrenia [1-4]. In addition to astrocytes, S100B is released from many cell types, such as, adipocytes, chondrocytes, cardiomyocytes and lymphocytes [5-13]. S100B can act in a paracrine and autocrine manner, where low concentrations regulate proliferation and differentiation of neurons and glia. S100B also acts as a neurotrophic factor, regulating dopaminergic and glutamatergic synaptic function, in addition to synaptogenesis [1,2]. In contrast, excessive levels of S100B promote the expression of inducible nitric oxide synthase or pro-inflammatory cytokines causing neuronal dysfunction and apoptosis. In S100B knockout mice, increased brain-derived neurotrophic factor and decreased noradrenaline further suggest a role for S100B in regulating the levels of neurotrophic factors and neurotransmitters [4]. Studies have also shown that copy number variations in S100B as well as polymorphisms in its gene and promoter region are associated with altered S100B protein levels in schizophrenia [14-21]. In addition, polymorphisms in the receptor for S100B, RAGE (receptor for advanced glycation end products) have been linked with schizophrenia, as have increased levels of the soluble version of RAGE [22,23].

At the protein level, findings suggest that S100B is increased in schizophrenia, and that these protein levels are correlated with medication, gender, age and illness severity [16,23-44]. Importantly, astrocytes express dopamine receptors (DA2Rs) and antipsychotic medications regulate the cellular release of S100B [45-48]. Of interest, S100B is also expressed in immune cells (including T cells and natural killer cells) and in adipocytes suggesting it may have a role in altered immune response and metabolic activity in schizophrenia [2,5-13]. Indeed, findings have suggested that altered S100B levels in schizophrenia may be due to metabolic disorder, visceral obesity, diabetes and/or immune dysfunction in this illness [6,8,9,34,49,50]. Here, the serum levels of S100B in patients diagnosed with schizophrenia was investigated and relationships between age, gender, illness severity, type of medication, treatment time and various metabolic factors, was examined. In this study, focus was given toward patients treated with clozapine, which has been previously shown to increase the incidence of hyperglycaemia, diabetes and metabolic syndrome [51]. Moreover, stratification of patients and control groups into male and female subpopulations were performed to investigate the impact of body mass index (BMI) on levels of S100B.

## Methods

### Study population

Ethical approval for this study was obtained from the St. Vincent’s Hospital Fairview Ethics Committee, Dublin, Ireland. Patients attending an urban mental health clinic who met the DSM-IV-TR (Diagnostic and Statistical Manual of Mental Disorders, 4th edition, Revised) [52] diagnostic criteria for schizophrenia were recruited. These patients were treated with clozapine (n = 97) or depot antipsychotic medication (n = 34). Healthy controls were recruited from the same urban area with matched demographics (n = 27). Informed, written consent was gained from all participants. Exclusion criteria for both groups included (i) other psychiatric and neurological diagnoses, (ii) a co-morbid diagnosis of substance abuse disorder and (iii) those with an IQ < 70. Healthy controls recruited did not have a significant physical disability or disease, or a personal or family history of psychiatric illness. While psychometric testing was not performed for individual participants, all participating subjects were educated to at least secondary school level in Ireland or equivalent. The age range of the healthy controls was 25–57 years of age (with mean age +/− standard deviation as 42.1 +/− 10.3) and for patients treated with clozapine medication was 22–78 years of age (with mean age +/− standard deviation as 42.5 +/− 12.2). Please see Table 1.

**Table 1:** Demographic data

**Demographic data**	**Patient group**	**Control group**
Subjects (Male:Famale)	97 (68:29)	27 (10:17)
Age (Range) (yrs)	42.5 ± 12.2 (22–78)	42.4 ± 10.3 (25–57)
Treatment duration (Range) (Yrs)	6.99 ± 4.64 (0.25-19)	n/a
Daily dose (Range) (mg/day)	432.1 ± 185.7 (125–900)	n/a
BPRS (18–126)	31 ± 9	n/a
SAN (0–125)	34 ± 18	n/a

Demographics of patients treated with clozapine and healthy control subjects. Brief Psychiatric Rating Scale (BPRS), Scale for the Assessment of Negative Symptoms (SANS). All values reported as mean +/− standard deviation. The range of values is shown in brackets, were indicated.

### Treatment conditions

The range of treatment duration for patients treated with clozapine was 0.25-19 years (with mean treatment duration +/− standard deviation as 6.99 +/− 4.64 years). The range of daily dose for clozapine medication was 125–900 mg per day (with mean daily dose +/− standard deviation as 432.1 +/− 185.7 mg per day). For depot medications (I.M.) the range of daily dose and the mean daily dose +/− standard deviation for (i) zuclopentixol was 50–450 per week and 133.3 +/− 119.9 mg per week, n =9; (ii) flupenthixol decanoate was 12.5-100 mg per week and 42.3 +/− 32.9 mg per week, n = 10; (iii) risperidone was 12.5-25 mg per week and 21.5 +/− 5.5 per week, n = 10; (iv) flupehazine decanoate was 25–50 mg per week and 41.7 +/− 14.4 per week, n = 3; and (v) haloperidol was 25–62.5 mg per week and 43.8 +/− 26.5 per week, n = 2. The chlorpromazine equivalent doses expressed as mean mg per day were as follows: clozapine 864 mg per day, zuclopentixol 133 mg per day, flupenthixol decanoate 423 mg per day, risperidone 172 mg per day, flupehazine decanoate 833 mg per day, and haloperidol 292 mg per day [53].

### Illness severity and clinical variables

To assess illness severity within the patient group, the Brief Psychiatric Rating Scale (BPRS) [54] and Scale for The Assessment of Negative Symptoms (SANS) [55] was administered by a trained clinical psychiatrist. The BPRS assesses severity of psychopathology in patients with schizophrenia, where values range between 18–126. The SANS scale was used for assessing and rating the severity of ‘negative symptoms’ in patients with schizophrenia, where patients scored between 0–125. To determine subject demographic and metabolic syndrome risk profile, clinical variables such as fasting cholesterol, HDL, LDL, triglycerides, and fasting glucose were determined for all participating subjects. Data including body mass index (BMI), blood pressure (BP), smoking status, a known diagnosis of diabetes and hypertensive treatment was obtained for all subjects. This enabled calculation of the Framingham score for all individuals within the groups studied. The Framingham score is a predictive score, showing the percentage risk of a cardiovascular event occurring for that participant in the subsequent 10 years [56].

### S100B analysis

All participating subjects were fasting for at least 12 hours prior to collection of blood sample for biochemical and S100B analysis. Blood samples obtained for S100B analysis were collected in monovette clotting activator serum tubes (Sarstedt, UK) and centrifuged to separate serum fraction. Serum samples were stored at −80°C until S100B analysis. S100B analysis was conducted by commercial ELISA assay (NKI-AVL, The Netherlands).

### Statistical analysis

All statistical analysis was performed using Prism 5 GraphPad Software package. Unless otherwise stated, statistical tests performed were either Student’s unpaired t-test (two-tailed) or one-way ANOVA (with Bonferroni post-hoc test). Individual statistical tests are described in figure legends. All data is shown as mean +/− SEM, where n = number of subject samples. The significance levels (or alpha levels) were set at p < 0.05*, p < 0.01** and p < 0.001***.

## Results

### Demographic data of patient cohort studied

In order to determine changes in S100B levels in patients diagnosed with schizophrenia, the demographics of control and patient cohorts were initially analysed. In addition, the patient cohort was scored for both negative and positive symptoms using the SANS and BPRS rating scales, respectively. A total number of 97 subjects diagnosed with schizophrenia, treated with clozapine, and 27 healthy control subjects were recruited (Table 1). The age range of the healthy controls was 25–57 years of age (with mean age +/− standard deviation as 42.4 +/− 10.3) and for patients treated with clozapine medication was 22–78 years of age (with mean age +/− standard deviation as 42.5 +/− 12.2). No statistical difference was found in age between these two groups (p = 0.962, Student’s unpaired t-test, two-tailed). While the male:female ratio was 57% for the patient group, this ratio was 41% for the control group. The BPRS and SANS rating for the patient group was 31 +/− 9 and 34 +/− 18, respectively (Table 1). This data indicates a typical patient population treated with clozapine displaying both positive and negative symptoms*.*

### Metabolic parameters altered in clozapine treated patient group

Cardiovascular disease is the leading cause of natural death in patients with schizophrenia [57]. Furthermore, it has been reported that patients diagnosed with schizophrenia have abnormal metabolic parameters prior to treatment with psychotropic medications [58]. In addition, previous studies show that clozapine increases the incidence of hyperglycaemia, diabetes and metabolic syndrome [51]. Here, a number of cardiovascular risk factors were investigated for both patient and control groups. The fasting glucose levels found in the patient group (5.52 +/− 0.09 mMol/L) compared to control group (5.06 +/− 0.16 mMol/L) was significantly raised (p = 0.0167, Student’s unpaired t-test, two-tailed) (Table 2). Furthermore, statistically significant abnormalities in Triglyceride (2.33 +/− 0.13 mMol/L vs. 1.11 +/− 0.12 mMol/L) (p = 0.0001, Student’s unpaired t-test, two-tailed) and HDL (1.07 +/− 0.03 vs. 1.41 +/− 0.11 mMol/L) (p = 0.0001, Student’s unpaired t-test, two-tailed) levels were observed between patient and control groups (Table 2). Notably, the levels of LDL (3.03 +/− 0.11 vs. 3.20 +/− 0.18 mMol/L) (p = 0.4579, Student’s unpaired t-test, two-tailed) and total cholesterol (5.08 +/− 0.13 vs. 5.11 +/− 0.16 mMol/L) (p = 0.9117, Student’s unpaired t-test, two-tailed) were not different between patient and control groups. These abnormal triglyceride and HDL levels likely predispose the patient group to an increased risk of cardiovascular and cerebrovascular disease. In addition, this data is in keeping with previous observations that clozapine may alter metabolic parameters [51].

**Table 2:** Biochemical data

**Biochemical data**	**Patient group**	**Control group**	**Significance**
Fasting glucose	5.52 ± 0.09	5.06 ± 0.16	0.0167*
Triglycerides	2.33 ± 0.13	1.11 ± 0.12	0.0001***
HDL	1.07 ± 0.03	1.41 ± 0.11	0.0001***
LDL	3.03 ± 0.11	3.20 ± 0.18	0.4579
Total cholesterol	5.08 ± 0.13	5.11 ± 0.16	0.9117

Biochemical data of patients treated with clozapine (n = 97) and healthy control subjects (n = 27). All biochemical values expressed as mMol/L. Statistical analysis was performed using Student’s unpaired T test (two-tailed). The significance levels (or alpha levels) were set at p < 0.05* and p < 0.001***.

### Clozapine treated patient cohort display increased BMI

Given the observations that metabolic parameters were altered in the patient cohort (Table 2) and that previous evidence has linked clozapine to weight gain [59], further analysis on cardiovascular risk was determined. In the current study, the Framingham Score, a prediction of a cardiovascular event occurring over the next 10 years, was calculated in the patient population analysed. No significant difference in the Framingham Score between patients and controls (7.01 +/− 0.62% vs. 5.31 +/− 0.89%) (p = 0.1797, Student’s unpaired t-test, two-tailed) was observed. In addition, an approximate 2-fold increase was observed in smoking rate between patient (61%) and control (30%) groups (Table 3). This finding has been well documented in patients with schizophrenia [60]. The data also showed a greater than 100-fold increase in diagnosis of type 2 diabetes within the patient group (6%) compared to control group (0.04%) was noted (Table 3). Importantly, a statistically significant difference in BMI (32.45 +/− 0.62 vs. 27.53 +/− 0.86) between patient and control groups (p = 0.0002, Student’s unpaired t-test, two-tailed) was observed (Table 3). This increase in BMI for the patient group is in concordance with the previous known association between patients treated with clozapine medication and subsequent weight gain [59].

**Table 3:** Cardiovascular disease risk factor and lifestyle data

**CVD Risk factors**	**Patient group**	**Control group**	**Significance**
Smoking status (+)	58/95 (61%)	8/27 (30%)	~2 fold increase
Diabetes mellitus (+)	6/95 (6%)	1/27 (0.04%)	>100 fold increase
Body Mass Index (BMI)	32.45 ± 0.62	27.53 ± 0.86	0.002***
Hypertension treatment	24/95 (25%)	6/27 (22%)	no change
Systolic blood pressure	123 ± 1.5	122 ± 0.16	0.5675
Total cholesterol	5.08 ± 0.13	5.11 ± 0.16	0.9117
Framingham (%)	7.01 ± 0.62	5.31 ± 0.89	0.1797

Cardiovascular disease risk factors data and Framingham-10-year (%) risk of cardiovascular disease incident of patients treated with clozapine (n = 97) and healthy control subjects (n = 27). Data expressed as mean ± SEM. Statistical analysis was performed using Student’s unpaired T test (two-tailed). The significance levels (or alpha levels) were set at p < 0.001***.

### The serum levels of S100B in patients treated with clozapine and healthy control subjects

**Figure 1:**
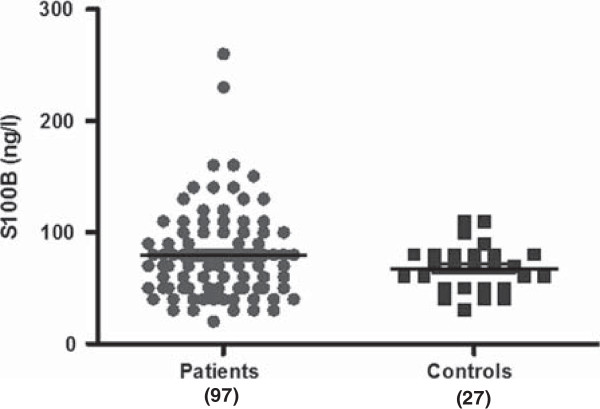
**S100B concentration for patients treated with clozapine and healthly control subjects.** Data shows S100B concentration for patients treated with clozapine (79.48 +/− 4.04 ng/L, n = 97) and healthy control subjects (67.78 +/− 4.01 ng/L, n = 27). The data showed no statistical significant difference in S100B serum concentration between patients treated with clozapine and control groups (p = 0.1438, Student’s unpaired t-test, two-tailed). All statistical analysis was performed using Student’s unpaired T test (two-tailed). Number of subjects in parenthesise (patients:controls).

A number of previous studies have suggested that the levels of S100B protein are altered in patients with schizophrenia, with the general observation that S100B levels are increased [16,19,29,61-63]. While some controversy exists, studies suggest that medication status may alter S100B concentrations [25,31,41,43,44]. Here, a study to further investigate the role of S100B in schizophrenia was conducted comparing patients treated with clozapine and healthy controls. The data showed no statistical significant difference in S100B serum concentration between patients treated with clozapine (79.48 +/− 4.04 ng/L, n = 97) and control groups (67.78 +/− 4.01 ng/L, n = 27) (p = 0.1438, Student’s unpaired t-test, two-tailed) (Figure 1). These results suggest that S100B serum concentration in patients with schizophrenia, treated with clozapine, are not significantly altered when compared to healthy controls, when analysing the whole sample population.

### S100B serum concentration is not related to age, treatment time or illness severity

**Figure 2:**
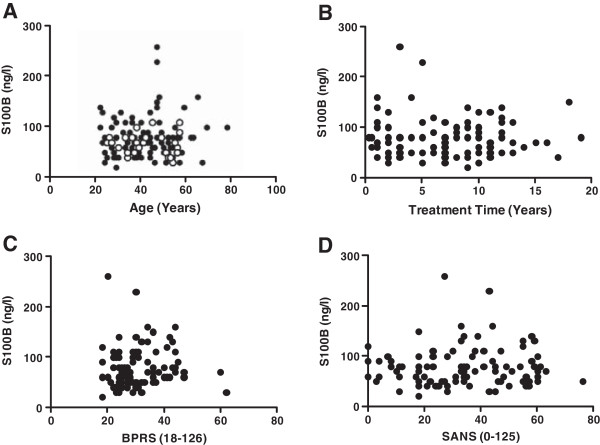
**S100B concentration in patients treated with clozapine.** S100B concentration plotted against (**A**) age, (**B**) time on clozapine treatment in years, (**C**) BPRS and (**D**) SANS. No notable correlations between S100B concentration any of these parameters in patient group were observed: age (Pearson’s correlation; r = −0.06026; p value (two tailed) = 0.7653 for control, Pearson’s correlation; r = 0.09035; p value (two tailed) = 0.3763 for clozapine treated patients), treatment time (Pearson’s correlation; r = −0.02633; p value (two tailed) = 0.7980), acute illness severity (BPRS) (Pearson’s correlation; r = 0.04655; p value (two tailed) = 0.649), negative symptoms illness severity (SANS) (Pearson’s correlation; r = 0.1874; p value (two tailed) = 0.8547), where the alpha value = 0.05. White circles (controls), black circles (patients).

Previous reports have suggested a correlation between the levels of S100B and age [39,61-63], antipsychotic medication [31,33,34,41-44], illness severity and symptoms [40,43]. However, these reports have in best part been conflicting. Thus, to further investigate these discordant findings, the age, treatment time and illness severity of participating subjects was recorded and plotted against S100B serum concentration. The data showed no significant correlation between the levels of S100B and age (Pearson’s correlation; r = −0.06026; p value (two tailed) = 0.7653 for control, Pearson’s correlation; r = 0.09035; p value (two tailed) = 0.3763 for clozapine treated patients) (Figure 2A), treatment time (Pearson’s correlation; r = −0.02633; p value (two tailed) = 0.7980) (Figure 2B), acute illness severity (BPRS) (Pearson’s correlation; r = 0.04655; p value (two tailed) = 0.649) (Figure 2C) or negative symptoms illness severity (SANS) (Pearson’s correlation; r = 0.1874; p value (two tailed) = 0.8547) (Figure 2D), where the alpha value = 0.05.

### Effects of medication on S100B serum concentration

**Figure 3:**
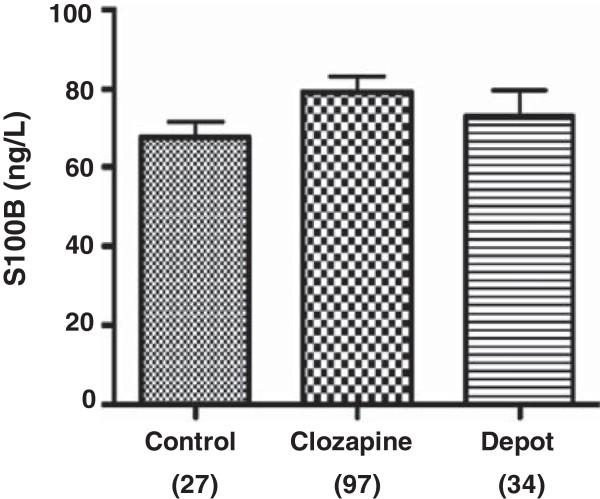
**Levels of S100B concentration in patients treated with depot medications.** Graph shows S100B concentration for patients treated with clozapine and those treated with depot medication compared to healthy control subjects. No statistical significance was observed between the groups. Number of subjects in parenthesise. Patients treated with depot medications include those treated with zuclopentixol (n =9), flupenthixol (n = 10), risperidone (n = 10), flupehazine (n = 3) and haloperidol (n = 2).

While some studies have reported the levels of S100B to be altered dependent on medication, these reports have been contradictory [31,33,35,41-44]. Furthermore, little is known about the direct effects of antipsychotic medication on glial cells within the central nervous system. To determine whether choice of antipsychotic medication effects the levels of S100B, this study also compared patients treated with clozapine (n = 97) and those treated with depot antipsychotic medications comprising zuclopentixol (n =9), flupenthixol decanoate (n = 10), risperidone (n = 10), flupehazine decanoate (n = 3) and haloperidol (n = 2). The results showed no statistical significant difference between clozapine treated patients (79.48 +/− 4.04 ng/L, n = 97), depot antipsychotic treated patients (73.08 +/− 6.64 ng/L, n = 34) and healthy controls (67.78 +/− 4.01 ng/L, n = 27) (p = 0.2706, clozapine treated patients and antipsychotic treated patients vs. control, one-way ANOVA and Bonferroni post-hoc test) (Figure 3). Taken together these data suggest that choice of antipsychotic medication has no significant effect on S100B serum concentration in patients with schizophrenia.

### S100B levels are elevated in female patients with schizophrenia

**Figure 4:**
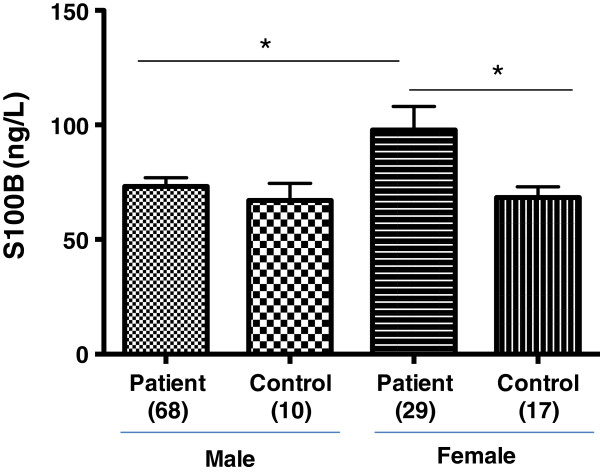
**S100B concentration in male and female patients treated with clozapine and healthy control subjects.** Data shows significant statistical difference in S100B concentration between male (73.24 +/− 3.70 ng/L, n = 68) and female (97.78 +/− 10.34 ng/L, n = 29) patients treated with clozapine (p = 0.0101, one-way ANOVA and Bonferroni post-hoc test). S100B concentration in female patients showed significant statistical significance compared to female controls (68.24 +/− 4.72 ng/L, n = 17) (p = 0.0101, one-way ANOVA and Bonferroni post-hoc test). No statistical difference was observed between male patient group and male control group (67.00 +/− 7.61 ng/L, n = 10) (as determined by one-way ANOVA and Bonferroni post-hoc test) (Figure 4). Data represented as mean +/− SEM. Number of subjects in parenthesise.

Given that previous studies have suggested that the levels of S100B may be dependent on gender [62-64], in the current study these levels were examined in male and female healthy control and patient groups. The data showed a statistically significant increase in levels of S100B in female patient group (97.78 +/− 10.34 ng/L, n = 29) compared to male patient group (73.24 +/− 3.70 ng/L, n = 68) (p = 0.0101, one-way ANOVA and Bonferroni post-hoc test) (Figure 4). Additionally, a statistically significant difference was found in S100B serum concentration between female patients and female controls (68.24 +/− 4.72 ng/L, n = 17) (p = 0.0101, one-way ANOVA and Bonferroni post-hoc test) (Figure 4). In contrast, no statistical difference was observed between male patient group and male control group (67.00 +/− 7.61 ng/L, n = 10) (as determined by one-way ANOVA and Bonferroni post-hoc test) (Figure 4). The data is supportive of previous studies indicating the levels of S100B differ in male and female genders. Studies have suggested that adipocytes release S100B, in addition to astrocytes. Furthermore reports show that insulin reduces the levels of S100B in adipocytes and astrocytes [10]. Of interest, overweight, visceral obesity and insulin resistance may be correlated with levels of S100B in schizophrenia [9]. Therefore, in addition to S100B being a marker for aberrant astrocyte function, it may also be associated with altered adipocyte cellular function.

### Levels of 100B correlate with BMI in female patients with schizophrenia

**Figure 5:**
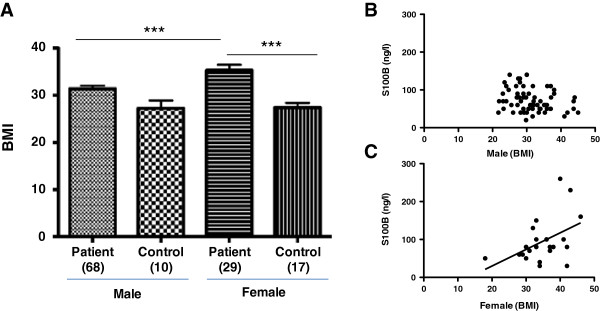
**S100B concentration in female patients treated with clozapine and healthy control subjects.** (**A**) Data shows significant statistical difference in BMI levels between male (31.37 +/− 0.69, n = 68) and female (35.31 +/− 1.16, n = 29) patients treated with clozapine. BMI levels in female patients also showed significant statistical significance compared to female controls (27.38 +/− 1.01, n = 17) and male controls (27.24 +/− 1.69, n = 10) (p = 0.0001, male patients and female controls vs. female patients, one-way ANOVA and Bonferroni post-hoc test). Data represented as mean +/− SEM. Number of subjects in parenthesise. Correlation between S100B concentration and BMI for (**B**) male and (**C**) female patients treated with clozapine. A significant correlation was observed for S100B concentration and BMI in female patients treated with clozapine (Pearson r = 0.4868; p value (two tailed) = 0.0117). Number of subjects in parenthesise.

To further examine if metabolic factors are associated with increased serum levels of S100B in females, a correlation between BMI and levels of S100B were examined in male and female patients. As indicated above, the data showed a statistically significant difference in BMI (32.45 +/− 0.62 vs. 27.53 +/− 0.86) between patient and control groups (p = 0.0002, Student’s unpaired t-test, two-tailed) (Table 3). Further analysis showed that female patients (35.31 +/− 1.16, n = 29) displayed increased BMI compared to male patients (31.37 +/− 0.69, n = 68) and compared to female controls (27.38 +/− 1.01, n = 17) and male controls (27.24 +/− 1.69, n = 10) (p = 0.0001, male patients and female controls vs. female patients, one-way ANOVA and Bonferroni post-hoc test) (Figure 5A). In addition, S100B serum concentration plotted against BMI showed a statistically significant correlation (Pearson’s correlation; r = 0.4868; p value (two tailed) = 0.0117) in female patients with schizophrenia, treated with clozapine (Figure 5C) but was not correlated in the male patient group (Pearson’s correlation; r = −0.2395; p value (two tailed) = 0.0511) (Figure 5B). An increase in BMI in the female patient group compared to male patient group and control groups is likely associated increased adipose tissue and is in agreement with the hypothesis that S100B protein in serum is influenced by not only altered release from astrocytes but also due to release from adipocytes in schizophrenia.

## Discussion

In the current study 97 subjects diagnosed with schizophrenia, treated with clozapine, 34 patients treated with depot antipsychotic medications and 27 healthy control subjects were recruited. Fasting glucose levels, triglyceride and HDL were raised in the patients compared to healthy controls. As expected the patient group showed an increased percentage of subjects who smoked and increased numbers with a known diagnoses of type 2 diabetes compared to control. Patients diagnosed with schizophrenia also showed higher BMI levels compared to healthy controls. When comparing S100B levels, no statistical difference was found between patients and controls. The serum levels of S100B were also not correlated with age, treatment time or illness severity. Analysis of S100B serum concentrations in patients treated with the depot antipsychotic medications including zuclopentixol, flupenthixol, risperidone, flupehazine and haloperidol also showed no observable differences in S100B levels compared to control. Importantly, the data here showed that S100B levels were elevated in female patients with schizophrenia compared to male patients and to those female and male healthy control subjects. Of interest, BMI levels were also elevated in female patients compared to male patients and healthy control groups. A correlation analysis showed that levels of S100B increased with BMI in female patients with schizophrenia. Taken together, the data suggested that levels of S100B are altered in schizophrenia and these levels are also likely related to patients BMI, in addition to astrocyte dysfunction.

The question has emerged if S100B is a specific marker for astrocyte dysfunction in schizophrenia? The widespread cellular expression of S100B and the lack of disease specificity for this protein [34,49], has questioned if S100B is a sole marker for astrocytes [50]. Astrocytes have diverse roles including (i) direct communication of astrocytic end-feet with endothelial cells that allows astrocytes to control the blood–brain-barrier; (ii) uptake of neurotransmitters, such as glutamate at the synaptic cleft, that allow astrocytes to regulate synaptic transmission and excitoxicity; and (iii) release of transmitters, growth factors, and cytokine/chemokines, allowing astrocytes to regulate cellular communication, migration and survival, for example of neurons and lymphocytes [65]. It is significant that astrocytes also play a role in scar formation after CNS injury, thus having apparent opposing roles in physiology and pathophysiology. In disease, astrocytes have been suggested to play roles in a range of psychiatric, neurological and neurodegenerative disorders. Previous studies have focused on the role of S100B in astrocytes, and suggested that astrocyte dysfunction may increase release of S100B in schizophrenia. However, S100B is also released from many other cell types including adipocytes and a number of *in vitro* studies have shown that levels of S100B from these fat cells can be regulated by, for example, glucagon, adrenaline and insulin [10,66,67]. Moreover, chronic fasting, weight gain and diet have also been shown to influence serum levels of S100B in patients and animal models [67,68]. The findings that S100B and BMI levels are elevated in female patients compared to male patients and to controls, and that S100B and BMI levels correlated in female patients (but not male patients), are in line with the hypothesis that visceral fat and altered adipocyte function could be a mechanism explaining elevated levels of S100B in schizophrenia [9,69]. On a more cautionary note, these results equally suggest that S100B can no longer be considered as a sole marker of astrocyte dysfunction in brain disease given its widespread distribution.

Previous studies have suggested that S100B levels in patients treated with clozapine are increased [33], which in part is in agreement with the data in the current study showing raised levels of S100B in female patients treated with clozapine. The effects of clozapine are thought to be mediated primarily via antagonism at 5HT2AR and DA4R, with weak DA2R blocking activity [70]. Astrocytes express DA2Rs and activation of these receptors using apomorphine decreases the levels of S100B in these cells, via a signalling pathway that involves inhibition of adenyl cyclase [46]. Moreover, antipsychotic medications (such as clozapine, haloperidol and risperidone), which block DA2Rs, also decrease S100B levels [46,47]. Treatment of astrocytic C6 cells and oligodendrocytic OLN-93 cells with haloperidol and clozapine also decreases the levels of S100B [48]. This data is in contrast with elevated levels of S100B observed in patients diagnosed with schizophrenia [46]. However, contradictory to the hypothesis that astrocyte derived S100B levels are raised in schizophrenia, is the finding that chronic antipsychotic medication, such as haloperidol or olanzapine, reduce astrocyte numbers in Macaque Monkeys [71]. Recent data also demonstrated that insulin downregulates levels of S100B in adipocytes (in addition to astrocytes) suggesting that other cell types may determine the levels of S100B [9]. Interestingly, molecular links between S100B, DA2Rs and schizophrenia have been suggested where S100B has been shown to interact with the third cytoplasmic loop of the DA2R, and to enhance receptor signalling to ERK and inhibition of adenylate cyclase [45,72-74]. In S100B transgenic animals, there is a decrease in the expression of DA2R suggesting crosstalk between S100B and DA2R function [75]. Taken together, these studies support the S100B/DA2R protein complex as a molecular target for antipsychotic medications and possible aberrant S100B/DA2R-mediated signalling in schizophrenia.

## Conclusions

This current study showed that levels of S100B are raised in female patients diagnosed with schizophrenia and correlate with BMI, which is possibly linked to higher levels of release from adipocytes. A limitation of this study was the unequal distribution of genders among the two groups (controls *vs.* patients). While ANOVA tests were performed, these assumed the sample size were equal and thus may have been too liberal. Another limitation of this study is the direct comparison of S100B levels between patients on medication and drug naive patients with schizophrenia. Previous studies have however reported that levels of S100B in drug naive patients with schizophrenia are raised [32,42]. Notably, however the levels of S100B were not correlated with BMI in these reports. In this current study we also did not investigate if the levels of S100B were associated with genetic mutations reported in schizophrenia. While it may be possible that our findings are explained, in part, by pathogenetic mechanisms, further studies would be required to determine this possibility, for example by investigating the levels of S100B in siblings of patients with schizophrenia. Moreover, further analysis of molecules that are more specific to adipocytes (rather than S100B) would be worthy of investigation. In addition, cellular studies demonstrating the effects of antipsychotic medications on the release of S100B in adipocytes would be warranted. In closing, while a number of studies have demonstrated that levels of S100B are altered in schizophrenia, many of these reports are contradictory when suggesting that age, gender, medication and illness severity all have an impact (or not) on S100B levels. The study here suggested two important factors that may help unify these apparent contradictory findings. Firstly, that the serum S100B concentrations are likely influenced by metabolic activity. Secondly, that the levels of S100B are not solely dependent on astrocyte dysfunction and may involve altered fat cell (adipocyte) function. Thus, the data presented here supports previous studies that have suggested an association between metabolic syndrome, diabetes and immune response dysfunction in schizophrenia. The clinical implications of this study are two-fold, firstly that S100B levels may no longer be considered as a biomarker that is dependent on neurological function alone, and secondly, that regulating metabolic dysfunction in schizophrenia may represent a novel drug target.

## Competing interests

The authors declare that they have no competing interests.

## Authors’ contributions

KKD and JT conceived the idea. KKD and KOC designed the methodology of this study. KOC was the major contributor in subject recruitment, in clinical and diagnostic assessments, in gathering experimental data and in conducted all the statistical analyses. KKD and KOC were the major contributors in the writing of the manuscript. All authors have approved the final manuscript.

## Pre-publication history

The pre-publication history for this paper can be accessed here:

http://www.biomedcentral.com/1471-244X/13/146/prepub
